# Fine Tuning of Electrical Transport and Dielectric Properties of Epoxy/Carbon Nanotubes Composites via Magnesium Oxide Additives

**DOI:** 10.3390/polym11122044

**Published:** 2019-12-09

**Authors:** Povilas Bertasius, Darya Meisak, Jan Macutkevic, Polina Kuzhir, Algirdas Selskis, Elena Volnyanko, Juras Banys

**Affiliations:** 1 Vilnius University, Sauletekio av. 3, LT-001257 Vilnius, Lithuania; pov.bertasius@gmail.com (P.B.); dariameysak@gmail.com (D.M.); juras.banys@ff.vu.lt (J.B.); 2 Institute for Nuclear Problems, Belarusian State University, 220006 Minsk, Belarus; polina.kuzhir@gmail.com; 3 Institute of Photonics, University of Eastern Finland, Yliopistokatu 7, FI-80101 Joensuu, Finland; 4 Center for Physical Science and Techology, Sauletekio Ave. 3, Vilnius, Lithuania; algirdas.selskis@ftmc.lt; 5 Metal Polymer Research Institute of the National Academy of Sciences of Belarus, Kirova str. 32a, 246050 Gomel, Belarus; volnianko@mail.ru

**Keywords:** epoxy, carbon nanotubes, dielectric permittivity, DC conductivity, transport

## Abstract

The dielectric properties of epoxy/MWCNT (multi-walled carbon nanotubes)/MgO hybrid composites with a fixed MWCNT amount of 0.12 vol.% (0.2 wt.%) and varying MgO concentrations up to 3 vol.% were investigated in broad frequency (20–40 GHz) and temperature (20–500 K) ranges. The composites with up to 2 vol.% MgO nanoparticles concentration showed a significant increase of DC conductivity in relation to their non MgO-containing counterparts. The optimal content of MgO was found, i.e., 0.46 vol.%, which gave up to 2.5 orders of magnitude larger DC conductivity than those of the samples prepared without MgO additives. Using various amounts of MgO, it is possible to predictably vary the broadband electromagnetic properties of the composites, even entirely eliminating the electrical percolation. Electrical transport at different temperatures can be substantially controlled by the addition of given amounts of MgO. The broadband properties are discussed in terms of the distribution of relaxation times, which are proven to be an effective, noninvasive, and simple tool for checking composite fabrication issues, such as the distribution of MWCNT aggregates within the epoxy matrix.

## 1. Introduction

Multifunctional composites are becoming ever more common and essential in modern industrial and commercial applications due to their being lightweight, flexible, and electrically conductive polymers for electromagnetic coatings. If an insulating polymer matrix is filled with a sufficient concentration of electrically conductive particles, the electrical percolation effect occurs, raising the conductivity sharply. Generally, it is desirable that the concentration at which the percolation appears is as low as possible, since this makes the composite less expensive and the overall mechanical performance better [1,2]. Carbon nanotubes (CNTs) [3] have been identified as a suitable filler for composites owing to their unique thermal, electrical, mechanical properties, and especially because of their extremely high length:diameter (i.e., aspect) ratio [1]. Commonly reported percolation thresholds for composites containing randomly distributed multi-walled CNTs (MWCNTs) in an epoxy resin matrix are between 0.01 wt.%–1 wt.%, depending on the preparation method, diameter, and the quality of the MWCNTs, functionalization of the MWCNTs surface, and age of the masterbatch among other parameters [1,2,4,5]. 

The huge surface area of the CNTs creates strong Van der Walls interactions between individual particles. During the preparation of CNT/polymer composites, due to the Van der Walls interactions, CNTs form extensive close-packed aggregates inside a low viscosity polymer matrix. Solution mixing is the most popular composite preparation method. To achieve the percolation threshold below 0.5 vol.% with this method, the perfect dispersion of CNTs is needed. In other words, the CNT aggregates have to be broken down with additional processes like ultrasonification, which itself might be destructive to the CNTs [2]. Residual aggregation shall remain, however, since it is important for providing good interconnection within the percolated network [4]. To achieve the lowest percolation threshold, CNTs need to be spaced as far apart as possible, while still being interconnected by conductive pathways. 

Nowadays, various porous composites filled with diverse inclusions attract attention due to their enhanced electromagnetic absorption, mechanical, and thermal properties [6,7,8,9,10], along with their low density. Indeed, huge electromagnetic absorption, as well as improved elastic and thermal properties are observed in epoxy composites with 3D copper nanowires and an annealed graphene aerogel framework [6]. To further increase performance, one of the appealing possibilities is to combine two or more fillers, and to exploit the synergetic effects between them. For example, many successful attempts to functionalize CNTs with an MgO coating [11,12,13,14,15,16,17], and to insert them into a polymer matrix [18,19,20,21], have been published. In order to attach MgO nanoparticles to the surface of CNTs, hydroxyl or carboxyl groups on the CNT surface are necessary [11,12,13,14,15,16,17,18,19]. This type of functionalization appears naturally if CNTs are in contact with oxygen, and additional extensive treatment of CNTs surface is unnecessary. The inorganic MgO nanoparticles can act as a spacer on the surface of CNTs which disallows the formation of Van der Walls bonds between CNTs, and they have been reported to prohibit the formation of CNT aggregates [13,18,20]. Thus, MgO nanoparticles can be used to control the CNT distribution. However, synergistic effects between MgO and CNT nanoparticles have not investigated to date.

The aim of this work is to find synergy between MgO nanoparticles and CNTs in the electrical properties of epoxy resin composites over a wide frequency range. Epoxy resin has excellent compatibility with CNTs owing to its low viscosity, and for this reason it was chosen as a host. 

## 2. Materials and Methods

### 2.1. Materials

The MWCNTs were grown by the СVD method as described in [21]. The MWCNTs used in this work had a diameter of 20–40 nm and length of 0.5–200 µm. Commercially available MgO nanoparticles were used for composite preparation (US Research Nanomaterials, Houston, TX, USA) [22]. The MgO nanoparticles had a mean particle size of 60 nm, density of 3.58 g/cm^3^, and specific surface area of 45 m^2^/g. The matrix used was EpikoteTM 828 epoxy resin, which is characterized by room temperature viscosity of 10–12 Pa, density of 1.16 g/cm^3^, and epoxy group content of 5340–5500 mmol/kg.

### 2.2. Preparation

Firstly, the MWCNTs were dispersed in ethanol for 30 min. The MWCNTs were then additionally dispersed using an ultrasonic bath for 1 h. The resulting mixture was combined with epoxy resin and underwent ultrasonication by ultrasonic dip for 2 h at 80 °C, whereby the ultrasonic power was 80 W. MgO was separately dispersed in ethanol and the solution was treated in an ultrasonic bath for 1 h, and then added to the MWCNT/epoxy mixture before being ultrasonificated once more by probe for 2 h. Consequently, the mixtures were placed under 50 °C for 2 days, so that ethanol would fully evaporate, and then the triethylenetetramine (TETA) hardener [23] was added (in a ratio of 1:10 with respect to the epoxy resin), and mechanically mixed for several minutes before the final products were poured into molds and left to harden for 24 h. Finally, the hardened composites were heated for 2 h at 100 °C and taken out of the molds.

**Figure 1:**
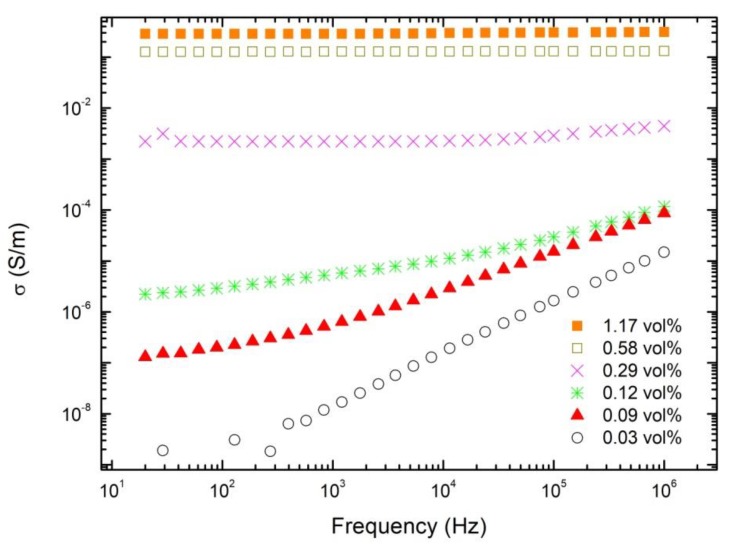
Frequency dependence of the electrical conductivity for epoxy resin composites with MWCNT inclusions.

Each produced composite had 0.12 vol. % (0.2 wt.%) MWCNTs concentration, which is close to the percolation threshold in our investigated MWCNT/epoxy composites (without MgO) (Figure 1). 

Six sets of samples were prepared with different MgO concentrations: 0, 0.25, 0.46, 1, 2, 3 vol.% (0, 0.8, 1.4, 3, 5.9, 8.7 wt.%, respectively). The surface morphology and microstructure were studied by scanning electron microscopy (SEM) using a Helios NanoLab 650 microscope. Several samples were investigated by SEM for the same MgO concentration.

### 2.3. Broadband Measurements

The dielectric properties were measured using an LCR meter HP4284 A in a broad frequency range of 20 Hz–1 MHz and in a temperature interval 30–500 K. Silver paint was used to make the electrical contact. A closed cycle helium cryostat with constant temperature change rate of 0.5 K/min was used for cooling below room temperature, while home-made furnace was used for higher temperatures. The dielectric measurements in 1 MHz–3 GHz frequency range were done with the coaxial line method using the vector analyzer E8363. The measurements in 26–40 GHz range were performed with a waveguide spectrometer which includes the generator Р2-65 and the scalar network analyzer R2400. Rectangular shaped samples were investigated in frequency range 20 Hz–3 GHz, while at microwaves the sample has the form of the thin cylindrical rod with typical cross section area about 0.5 mm^2^. The electrical conductivity ($\sigma^{\prime }$) was calculated using the expression:

(1) $$\sigma^{\prime }=\omega \varepsilon_{0}\varepsilon^{\prime \prime }$$

## 3. Results and Discussion

### 3.1. Room Temperature Properties

**Figure 2:**
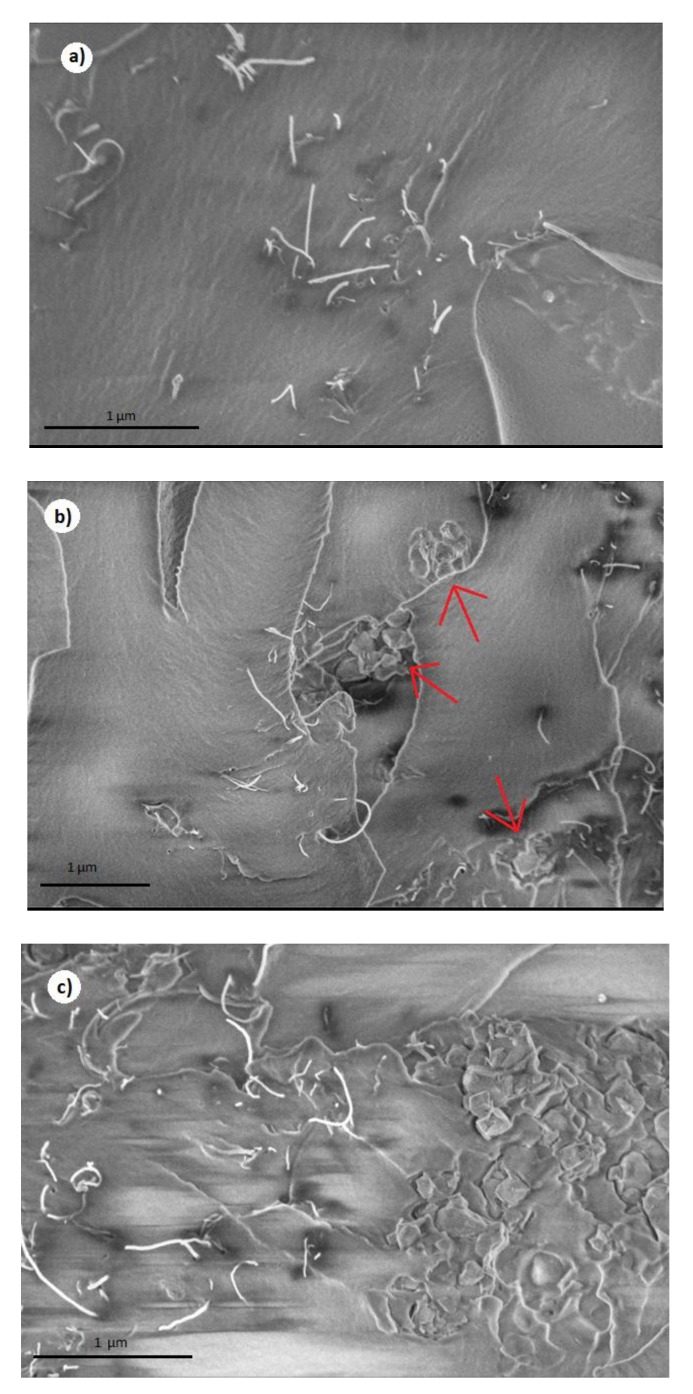
SEM micrographs of epoxy resin composites with 0.12 vol.% CNT and 0 (**a**), 0.25 (**b**), 3 (**c**) vol. % MgO content. Red arrows indicate small MgO aggregates.

SEM images of the prepared composites with 0.12 vol.% MWCNT and 0, 0.25, and 3 vol.% MgO are presented at Figure 2. The dispersed MWCNTs are clearly visible. MgO nanoparticles can also be seen as aggregates of various diameters in Figure 2b with 0.25 vol.% MgO around 500 nm diameter aggregates indicated by red arrows, in Figure 2c with 3 vol.% MgO wherein each aggregate more than 2 µm in diameter can be observed. It can be seen that a higher MgO content results in bigger MgO aggregates. Concerning MWCNTs, the cross-sectional area of their agglomerates can be identified to be the highest in composite with 3 vol.% MgO, and the smallest in the sample with 0.25 vol.%. Therefore, MgO nanoparticles have an impact on the dispersion of MWCNTs. The addition of MgO nanoparticles into epoxy resin decreases the glass transition temperature and makes the degree of crosslinking lower [24,25]. Moreover, the viscosity of epoxy resin becomes lowers, which increases the CNTs agglomeration during the solution mixing [4]. However, with very small MgO concentrations, the opposite effect can be observed. A possible explanation is that MgO nanoparticles act as a ‘milling’ agent during the sonification of the composite masterbatch, effectively grinding the aggregates into smaller sizes. 

**Figure 3:**
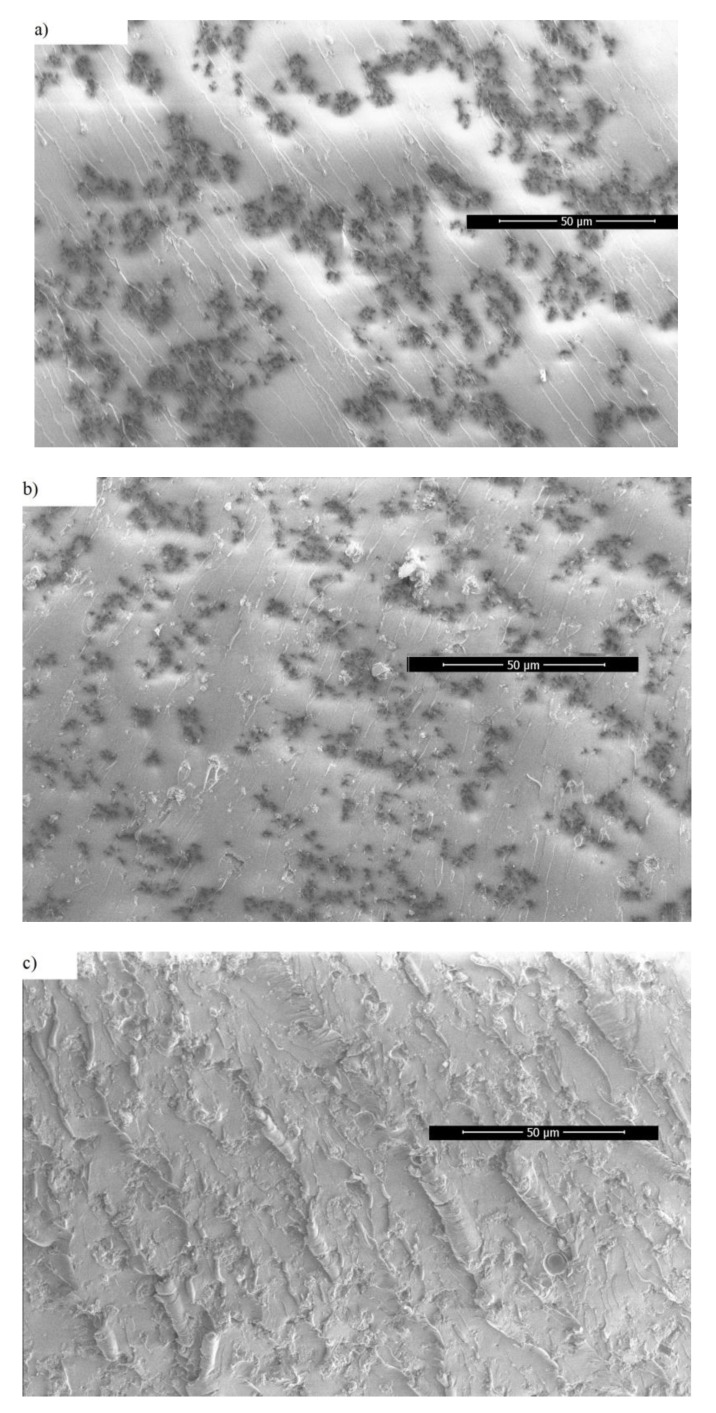
SEM micrographs (panoramic view) of epoxy resin composites with 0.12 vol.% CNT and 0.25 (**a**), 1 (**b**), 3 (**c**) vol.% MgO content. Red arrows indicate small MgO aggregates.

In order to see the macroscopic distribution of MWCNTs, the panoramic SEM of composites is presented in Figure 3 (MWCNT clusters are observed as black spots, which are confirmed by higher resolution SEM pictures). The hierarchical structure is clearly observed in composites with 0.25 vol.% MgO (Figure 3), in composites with 1 vol.% clusters of MWCNT are uniformly distributed, while in composites with 3 vol.% MgO, no macroscopic structure of the MWCNT is observed. This is in good agreement with previously reported results that the MWCNT clustering can decrease the percolation threshold value [26]. Smaller MgO clusters acts as separators of MWCNT clusters (Figure 2b) and support certain macroscopic structures of the MWCNT clusters (Figure 3a,b).

**Figure 4:**
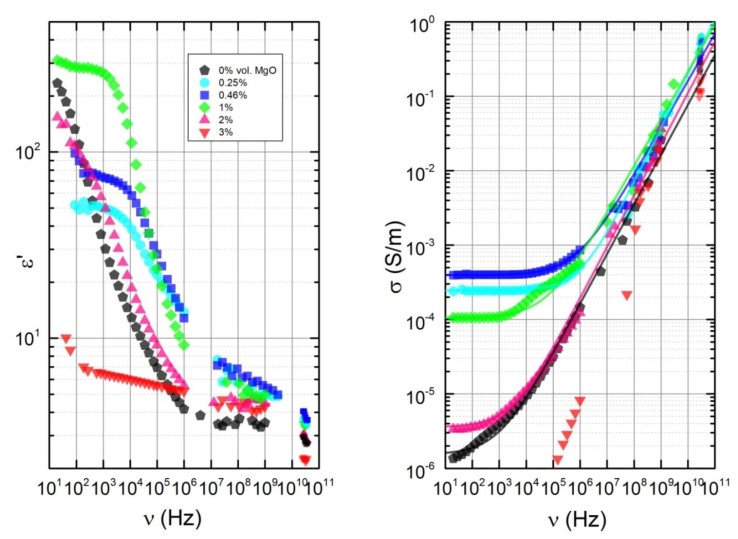
Frequency dependencies of the dielectric permittivity and the electrical conductivity at room temperature.

Frequency spectra of the dielectric permittivity ($\varepsilon^{\prime }$) and the electrical conductivity ($\sigma^{\prime }$) at room temperature for all the samples are presented in Figure 4. The shape of dielectric permittivity and conductivity spectra is strongly dependent on the MgO concentration. The differences in electrical properties of composites are clearly expressed at low frequencies (below 1 MHz), while at higher frequencies, electrical properties are less dependent on the MgO concentration. At low frequencies (below 1 kHz) the highest electrical conductivity is observed for the MgO concentration of 0.12 vol.%, while the dielectric permittivity is highest for 1 vol. %. Composites with 2% MgO and without MgO additions have very similar broadband electrical properties. The values of $\varepsilon^{\prime }$ and $\sigma^{\prime }$ for composites with 3 vol.% MgO inclusions are very small and resemble the dielectric properties of pure epoxy resin.

At low frequencies the frequency-independent conductivity, which coincides with the DC conductivity ($\sigma_{DC}$), is visible for all samples, except the one containing 3 vol.% MgO. The conductivity in such cases can be approximated using the Almond–West type power law:

(2) $$\sigma^{\prime }=\sigma_{DC}+A\omega^{s}$$

where A and s are parameters (0<s≤1) shown together with fitting errors in Table 1. The fitting curves are presented in Figure 4 as solid lines. The appearance of $\sigma_{DC}$ is an indication that an electrical percolation network is presented in the sample. The frequency at which the value of the conductivity σ(ω) deviates from the DC plateau is called the critical frequency $f_{cr}$. Close to the percolation threshold $p_{c}$, the critical frequency $f_{cr}$ is a function of the filler concentration p and a characterizing parameter t:

(3) $$f_{cr}\sim {\left(p-p_{c}\right)}^{-t}$$

From the observed $f_{cr}$ values, it can be determined that the use of MgO (concentrations from 0.25 vol.% to 2 vol.%) during the preparation, together with 0.12 vol.% MWCNTs, decreases the critical percolation concentration $p_{c}$. The electrical percolation threshold is the lowest for composites with 0.46 vol.% MgO inclusions because, for this particular case, the DC conductivity and critical frequency have the highest values. Further increasing the MgO concentration above 0.46 vol.% led to a decrease of $\sigma_{DC}$, while the sample with 3 vol.% MgO didn’t exhibit electrical percolation at all. The SEM micrographs in Figure 2 and Figure 3 can be used to explain these results. In composites with 0.25 vol.% MgO inclusions (which had a very similar $\sigma_{DC}$ to that of 0.46 vol.% MgO), the MWCNTs are better dispersed than in composites with 3 vol.% MgO inclusions because of the smaller aggregate sizes. This results in a much higher level of interconnectivity, and consequently electrical conductivity. The average distances between clusters and stand-alone MWCNTs in composites with 3 vol.% MgO inclusions are too high to achieve percolation, with huge aggregates also containing fewer and shorter splintered MWCNTs, which is important to achieve good interconnection. Additionally, huge MgO aggregates presented in high MgO content samples form an obstacle to prohibiting the formation of a percolation network.

### 3.2. Electrical Transport at Different Temperatures

**Figure 5:**
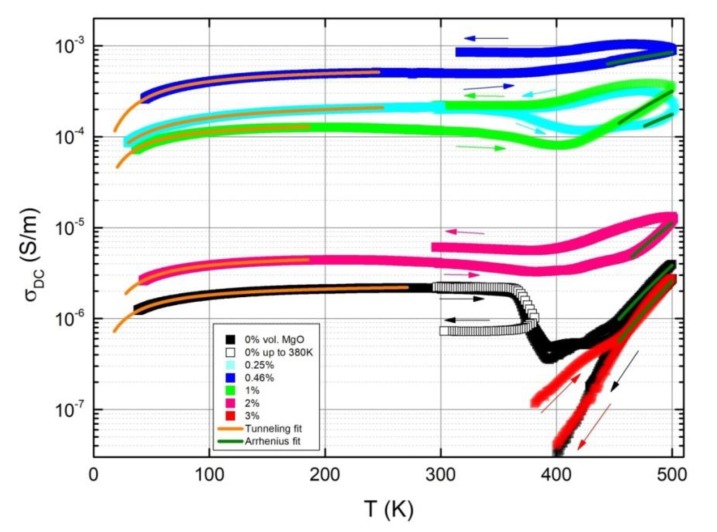
Temperature dependence of the DC conductivity. Arrows indicate the direction of heating and cooling. Solid lines are approximations according to Equations (4) and (7), and parameters are presented in Table 2 and Table 3, respectively.

To observe how the DC conductivity changes with temperature, electrical measurements over a broad temperature range of 30–500 K were performed. The temperature dependence of the DC conductivity is presented in Figure 5. Three different temperature regions can be separated: (a) below 250 K, the electrical conductivity increases with the temperature; (b) in a temperature range of 250–350 K, the electrical conductivity decreases when the temperature increases, and (c) above 350 K temperature the electrical conductivity again increases with temperature. Moreover, after annealing, the electrical conductivity exhibits pronounced hysteresis.

In a non-homogenous system of the polymer matrix and conductive filler, the main method of charge transfer is variable-range hopping and/or tunneling through an energy barrier when mean distances between conductive nanoparticles are up to several nanometers [27,28]. In the latter case, the DC conductivity can be expressed through temperature using the tunneling model [29]:

(4) $$\sigma_{dc}=\sigma_{0}\exp\left(\frac{-T_{1}}{T+T_{0}}\right)$$

where $T_{1}$ represents the energy required for an electron to cross the insulator gap between the conductive particle aggregates, $T_{0}$ is the temperature above which thermally activated conduction over the barriers begins to occur, and $\sigma_{0}$ is the pre-exponential factor. Equation (4) describes the temperature dependence of DC conductivity below 250 K for all composites very well, as can be seen in Figure 5. The parameters are listed in Table 2 together with fitting errors. Parameters $T_{0}$ and $T_{1}$ can be expressed by:

(5) $$T_{1}=8\varepsilon_{0}wA\beta_{0}{}^{2}/k$$

(6) $$T_{0}=2T_{1}/\pi \chi w$$

where $\chi ={\left(2mV_{0}\right)}^{0.5}/ћ$ and $\beta_{0}=4V_{0}/ew$, m and e being the electron mass and charge, respectively, $\varepsilon_{0}$ is the vacuum permittivity, $V_{0}$ is the potential barrier height, w is the interparticle distance (gap width), and A is the area of capacitance formed by the junction. If we assume that A is the cross-sectional of a MWCNT with diameter values between 20 and 40 nm, the values of $V_{0}$ and w are calculated according to Equations (5) and (6), and these are presented in Table 2. The values of $V_{0}$ can be seen as quite similar for all samples. The interparticle distance w mainly increases with MgO concentration, however, the minimal value occurs for 0.25 vol.%. While the increase of w has a negative impact on electrical conductivity, the experimental measurements with up to 2 vol.% MgO content shows a substantial increase of electrical conductivity. This might be explained as a higher level of interconnectivity becomes present between the MWCNTs inside the samples with up to 2 vol.% MgO characterized by a higher number of contact areas between MWCNTs, which would lead to an increase in the electrical conductivity. 

In a temperature range of 250–300 K, the DC conductivity decreases as temperature increases due to the heat-induced stretching and increase in the volume of the polymer matrix [28]. At high temperatures (above 400 K), the DC conductivity begins to increase again, which is the result of an onset of electrical conductivity in the polymer matrix. Therefore, above 400 K, DC conductivity is observed, even in the composite with 3 vol.% MgO, which is below percolation threshold. In the high temperature region, the Arrhenius equation is valid for DC conductivity [30]:

(7) $$\sigma =\sigma_{0}\exp(-E_{a}/kT)$$

where $\sigma_{0}$ is the pre-exponential factor and $E_{a}$ is the activation energy. The obtained parameters are listed in Table 3, wherein the activation energy of pure epoxy resin DC conductivity is 1.28 eV according to [28]. The total conductivity is the sum of the electrical conductivity inside the matrix and in the percolation network, since both of the ‘resistors’ are connected in parallel. However, this means that the activation energy of total conductivity and the matrix are not equal to each other. With up to 0.46 vol. % MgO, low activation energy values are observed, comparable to the potential barrier $V_{0}$ seen previously (see Table 2). This implies that the governing method of transport is electron tunneling, while in all other cases with much higher energy, it is mostly the conductance through the polymer matrix. 

Samples with up to 2 vol.% MgO showed hysteresis during a heating–cooling cycle, with slightly higher $\sigma_{dc}$ after annealing. However, for the sample without MgO a sharp break down of $\sigma_{DC}$ appears around 400 K during heating, indicating a destruction of the percolation network. After annealing at 500 K, the structure of these composites was permanently damaged, the value of σ became similar to that of the sample with 3 vol.% MgO, which had no percolation network. The composites became electrically nonconductive at room temperature after annealing. 

After annealing, the electrical conductivity also decreases for composites with 3 vol.% MgO inclusions. The impact of MgO inclusions on the temperature dependence of the conductivity can be explained by the significantly lowered threshold of the electrical percolation in hybrid MgO/MWCNT composites with up to 2 vol.% MgO. This is expected to be lower for composites with 0.46% MgO. Composites close to the percolation threshold are mostly unstable and their percolation network can be easily destroyed by annealing, while the properties of composites far above the percolation threshold are stable [31,32]. In the intermediate case, enhancement of the electrical properties is observed. In order to analyze the gradual percolation network breakdown, composites without MgO were annealed only up to 380 K, and a significant nonreversible decrease of DC conductivity was observed, although in this case the network did not break down entirely, as in the case of annealing up to 500 K. 

**Figure 6:**
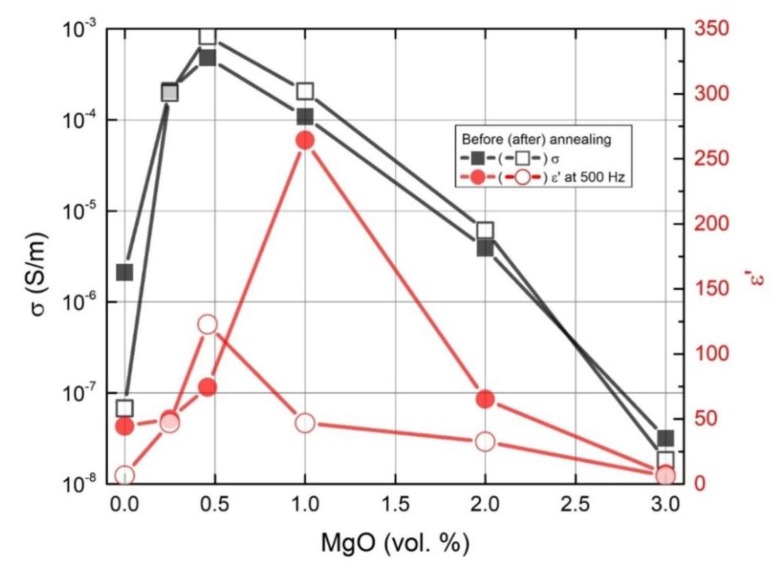
Electrical conductivity of composites with different MgO vol.% concentrations at room temperature, 129 Hz before, and after annealing at 500 K.

The results of $\sigma_{dc}$ and $\varepsilon^{\prime }$ at 500 Hz at room temperature for all composites are presented in Figure 6. The electrical conductivity was maximal at 0.5 vol.% MgO, while the dielectric permittivity at 500 Hz was minimal at 1 vol.% MgO before annealing and 0.5 vol.% after annealing.

To obtain more information from the dielectric measurements before and after annealing at 500 K, we calculated the complex impedance, in which real ($Z^{\prime }$) and imaginary ($Z^{\prime \prime }$) parts can be expressed as:

(8) $$Z^{\prime }=\frac{\varepsilon^{\prime \prime }}{{\varepsilon^{\prime }}^{2}+{\varepsilon^{\prime \prime }}^{2}}\frac{1}{\varepsilon_{0}\omega }$$

(9) $$Z^{\prime \prime }=\frac{\varepsilon^{\prime }}{{\varepsilon^{\prime }}^{2}+{\varepsilon^{\prime \prime }}^{2}}\frac{1}{\varepsilon_{0}\omega }$$

**Figure 7:**
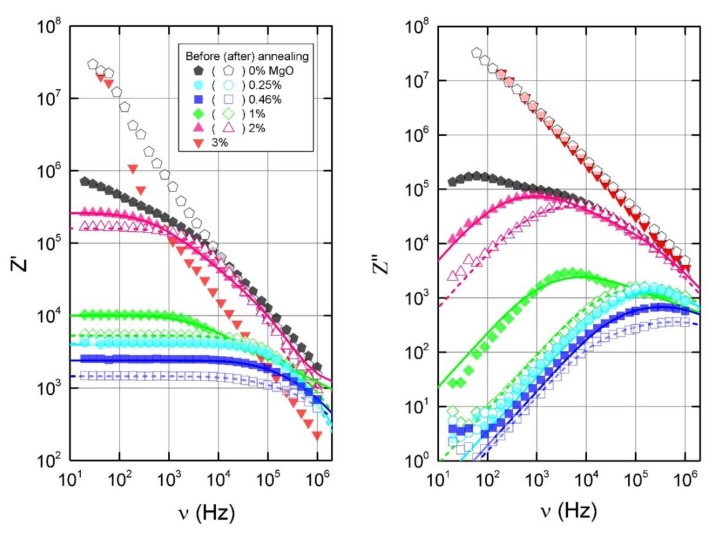
Frequency dependence of the complex impedance before and after annealing at 500 K.

The results at room temperature are presented in Figure 7. The real part of the impedance shows frequency independent values at the same frequencies at which $\sigma_{dc}$ is visible. The imaginary part shows maxima at various frequencies depending on the sample (except the sample with 3 vol.% MgO). After annealing at 500 K, the maxima for some samples shift to higher frequencies, except in the case composites with 0.25 vol.% of MgO, whereby the electrical properties remain stable after annealing. The frequency dependence of complex impedance is related to Maxwell–Wagner relaxation [33] and can be modeled with an equivalent circuit, for example, as the infinite circuit of RC connected in serial. The corresponding distribution of relaxation times (f(τ)) was obtained by the method described in [34] and resolves the integral equation when τ=RC:

(10) $$Z^{*}\left(\nu \right)=Z_{\infty }+\Delta Z\overset{\infty }{\underset{-\infty }{\int }}\frac{f\left(\tau \right)d\log\tau }{1+i\omega \tau }$$

**Figure 8:**
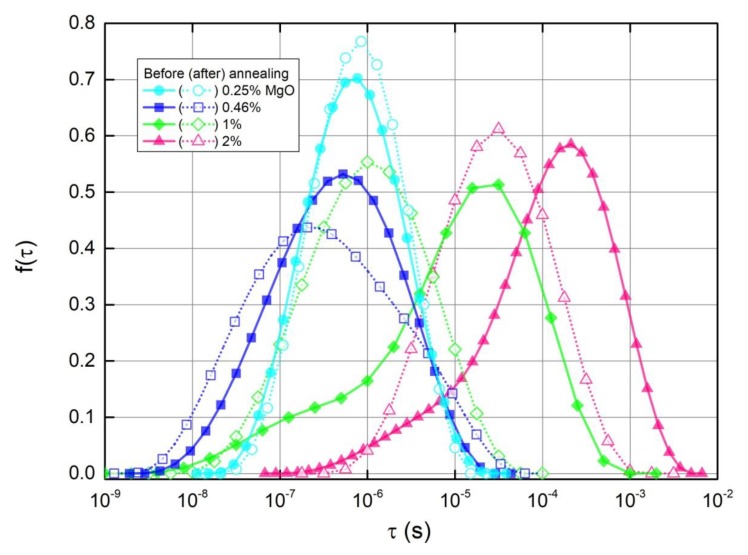
Distributions of relaxation times obtained from Equation (10) before and after annealing at 500 K.

The calculated distributions of relaxation times are presented in Figure 8. For the composite without MgO, the distribution could not be calculated since the imaginary part’s maximum position appears below the considered low frequency limit, however, the maximum can be reliably expected at the longest τ value seen in Figure 8. The distributions are symmetrical for all presented samples, except for composites with 1 vol.% and 2 vol.% MgO before annealing, which have ‘tails’ stretching into shorter relaxation times. After annealing, these ‘tails’ disappear and the distributions shift into shortest relaxation times. For composites with 0.25 vol.% MgO, there is almost no change after annealing, and the distribution of the composite with 0.46 vol.% MgO shifts into the shortest times only very slightly. It is known that smaller conductive clusters have shorter relaxation times, since relaxation is dependent on the capacitance, which is directly proportional to the cluster size. Shorter distances between conductive clusters also increases the conductivity, and consequently results in a shorter relaxation time. As a result, the shift to shorter relaxation times can be explained as a breakdown of conductive clusters into smaller ones, resulting in better dispersion inside the matrix. With increasing concentrations of MgO, there are more MWCNT aggregates, which can be broken down after annealing.

Considering the dielectric properties of percolative composites, the complex dielectric permittivity is related to the complex impedance

(11) $$\varepsilon^{*}=\frac{i}{\omega \varepsilon_{0}Z^{*}}\;\mathrm{OR}$$

(12) $$\varepsilon^{\prime }=\frac{Z^{\prime \prime }}{\varepsilon_{0}\omega ({Z^{\prime }}^{2}+{Z^{\prime \prime }}^{2})}$$

(13) $$\varepsilon^{\prime \prime }=\frac{Z^{\prime }}{\varepsilon_{0}\omega ({Z^{\prime }}^{2}+{Z^{\prime \prime }}^{2})}$$

Thus, the dielectric permittivity ε’ decreases with frequency at higher frequencies at which Z” > Z’ (ω > 1/τ_max_ (τ_max_ is the relaxation time at which f(τ) has the maximum). 

At low frequencies (Z” << Z’ and (or) ω < 1/τ_max_):

(14) $$\varepsilon^{\prime \prime }=\frac{1}{\varepsilon_{0}\omega Z^{\prime }}\approx \frac{\sigma_{DC}}{\varepsilon_{0\omega }}$$

(15) $$\varepsilon^{\prime }\approx \frac{Z^{\prime \prime }\sigma_{DC}{}^{2}}{\varepsilon_{0\omega }}$$

In this case, the behaviour of the dielectric permittivity at a fixed frequency, ω_fix_, correlates with the behaviour of the DC electrical conductivity only if Z” at low frequencies or f(τ) at long relaxation times remain the same (Figure 6, Figure 7, Figure 8). The dielectric permittivity can be frequency independent also only at low frequencies and if Z”~ω.

## 4. Conclusions

The dielectric properties of epoxy/MWCNT/MgO hybrid composites with a fixed MWCNTs amount of 0.12 vol.% and varying MgO concentrations up to 3 vol.% were investigated over broad frequency and temperature ranges. The composites with up to 2 vol.% MgO nanoparticles concentration showed a significant increase of electrical conductivity values over their non-MgO-containing counterparts. This proves that MgO nanoparticles work as agents, which up to 2 vol.% MgO promote better dispersion of MWCNTs inside the matrix during the simple preparation process via the solution mixing. Composites with the optimal 0.46 vol.% MgO concentration demonstrated up to 2.5 orders of magnitude larger electrical conductivity than samples prepared without MgO. Using 0 vol.%–3 vol.% of MgO, it is possible to predictably vary the dielectric properties of the samples, even entirely eliminating the electrical percolation for composites with 3 vol.% MgO inclusions. Furthermore, samples which were enhanced with MgO showed resistance to high temperature percolation network degradation, and even benefited from second annealing at up to 500 K, while in the case of pure MWCNT composites without MgO additives, a complete percolation network breakdown was observed at this temperature. The dielectric analysis was also found to be an effective tool for studying the distribution of nanoparticles in hybrid MWCNT/MgO epoxy composites, i.e., the shift to shorter relaxation times is a sign of the breakdown of conductive clusters into smaller ones, and thus a better dispersion inside the polymer matrix.

## Figures and Tables

**Table 1:** Approximations according to Almond–West type power law.

MgO Content (vol. %)	σ_DC_ (S/m)	*A* (S)	*s*
0	1.54 × 10^−6^	1.55 × 10^−^^8^	0.67
0.25	2.45 × 10^−^^4^	5.96 × 10^−^^9^	0.73
0.46	3.93 × 10^−^^4^	6.99 × 10^−^^8^	0.64
1	9.18 × 10^−^^5^	8.23 × 10^−^^8^	0.63
2	3.54 × 10^−^^6^	1.36 × 10^−^^8^	0.69

**Table 2:** Approximation parameters according to the tunneling model (4).

MgO (vol.%)	σ_0_ (S/m)	*T_1_* (K)	*T_0_* (K)	$V_{0}\;(\mathrm{meV})$	*w* (nm)
0	2.5 × 10^−6^	34.8	10.3	7.6	4.9
0.25	2.6 × 10^−^^4^	57.4	22.1	8.3	3.7
0.46	6.1 × 10^−^^4^	45.7	9.3	9.9	6.4
1	1.5 × 10^−^^4^	30.6	5.4	8.9	7.8
2	5.3 × 10^−^^6^	33.5	4.7	10.1	9.2

**Table 3:** Approximation coefficients according to the Arrhenius Equation (7).

MgO Content (vol. %)	$ln\left\{\sigma_{0},S/m\right\}$	$E_{a}/k,\;K,\;(\mathrm{eV}$
0	1.038	6751 (0.58)
0.25	7.391	713 (0.06)
0.46	4.737	1168 (0.1)
1	2.75	5417 (0.47)
2	1.904	6631 (0.57)
3	3.348	8090 (0.7)
